# The effectiveness of intra-articular vs subacromial corticosteroid injection for frozen shoulder: Study protocol for a randomized controlled trial

**DOI:** 10.1097/MD.0000000000019706

**Published:** 2020-04-17

**Authors:** Yanbiao Wang, Jing Gong

**Affiliations:** a Department of Orthopedics, Affiliated Hospital of Shandong Academy of Medical Sciences, Shandong First Medical University; b Department of Anesthesiology, The 960th Hospital of the People's Liberation Army Joint Logistice Support Force, Shandong, China.

**Keywords:** corticosteroid injection, frozen shoulder, intra-articular, randomized controlled trial, study protocol, subacromial

## Abstract

**Background:**

Intra-articular (IA) corticosteroid injection is a commonly used therapy for frozen shoulder (FS), but controversy still exists regarding the injection site with the best outcome. This randomized controlled trial is designed to determine whether corticosteroid injection into the subacromial space was not inferior to IA injection in patients with FS.

**Methods:**

This study will be a single-center, randomized, and double-blinded trial. Sixty patients who meet inclusion criteria will be randomized in a ratio of 1:1 to either subacromial injection or IA injection group. The outcome evaluations will be conducted at 4 time points (baseline, 4, 8, and 12 weeks after the injection) by an independent physical therapist. The primary outcome measure is visual analog scale for pain, whereas the secondary outcomes include Constant score, and shoulder passive range of motion including abduction, forward elevation, external rotation at the side, and internal rotation at the side.

**Discussion:**

This study has limited inclusion and exclusion criteria and a well-controlled intervention. This clinical trial is expected to provide evidence of proper site of corticosteroid injection for the treatment of FS.

**Trial registration:**

This study protocol was registered in Research Registry (researchregistry5368).

## 1 Introduction

Frozen shoulder (FS), a painful condition with gradual onset in the glenohumeral joint, is a common cause of shoulder disability.^[1]^ It has been reported the yearly prevalence of FS in general population to be 2.4/1000.^[2,3]^ FS is considered as a self-limited process from synovial inflammation to capsular fibrosis with three overlapping phases: the freezing phase (2–9 months), the stiffening phase (4–12 months), and the thawing phase (12–42 months).^[4]^ Typically, severe pain occurs mainly in the first 2 stages, and most patients can relieve pain and restore activity within 1 to 2 years after the onset of disease, but some residual joint stiffness may remain.^[5]^ Although the exact etiology have not yet been well understood, factors such as age, diabetes, autoimmune diseases, and trauma may associate with FS.^[6,7]^

Two most commonly used treatments for FS are physical therapy and local steroid injections. Combining injection with physical therapy may improve the efficacy in the treatment of shoulder stiffness.^[8]^ Triamcinolone acetonide is a well-known long-acting corticosteroid. Early treatment with local injection may reduce synovial inflammation, which leads to a decrease in pain perception and early acceleration of functional recovery. Several previous systematic reviews reported the effects of corticosteroid injections better than physiotherapy or placebo within 6 weeks, but effect on long-term follow-up is uncertain.^[9,10]^

However, various injection techniques, such as subacromial (SA) injection, intra-articular (IA) injection, and rotator interval injection are commonly used by orthopedic surgeons, primary care physicians, and rheumatism doctor. The argument on the best injection sites remains to be resolved. Some randomized controlled trials have reported similar effects between the IA and SA treatments,^[11,12]^ the other 2 randomized controlled trials claimed the superiority of IA injection up to 12 weeks for pain relieve and functional recovery compared to SA injection.^[13,14]^ With injection of 40 mg of triamcinolone and 2 mL 2% lidocaine, Kim et al found RI injection was superior to IA in the early phase of FS.^[15]^

Therefore, a prospective, randomized study will be designed to compare short-term outcomes between SA and IA corticosteroid injections in patients with primary FS. The aim of current study is to reveal which treatment modality is superior in terms of the visual analog scale (VAS) score for pain as well as functional outcomes, including constant score, and range of motion (ROM). We hypothesized that corticosteroid injection into the SA will provide similar clinical outcomes compared with IA.

## 2 Material and method

### 2.1 Study design

**Figure 1:**
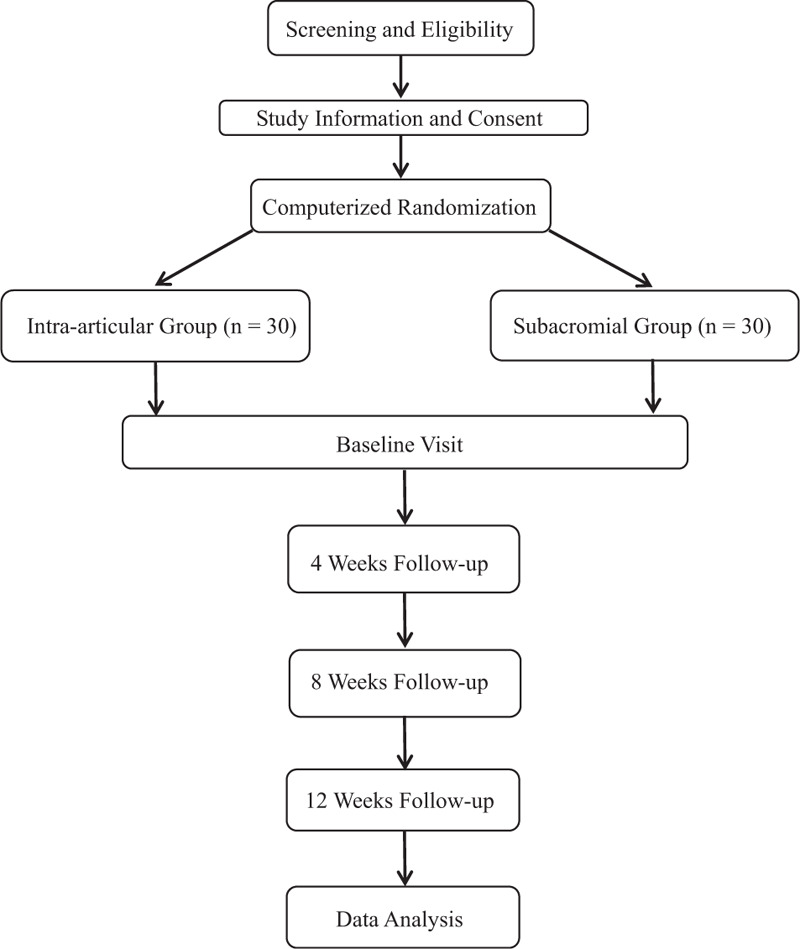
Flow diagram of the study.

This single center, double-blinded, randomized controlled trial is conducted in accordance with the Declaration of Helsinki principles. The study will be conducted in the Affiliated Hospital of Shandong Academy of Medical Sciences from March 01 2020 till February 28 2021. The study protocol was approved by local ethics committee board (BZSY-2020KYKTPJ-07) and subsequently registered in Research Registry (researchregistry5368). The flowchart of this trial is shown in Figure 1.

### 2.2 Recruitment and consent

All patients with FS will visit articular orthopedic outpatient at our hospital, where they will be invited to participate the study by treating surgeon. The surgeon will introduce the specific content of the trial and then answer all of patient's questions patiently. Meanwhile, a written information about our trial will be provided to the patient. Written informed consent will be required if the patient agrees to participate in the study. It is acceptable to withdraw from the study at any time because patient participation is voluntary.

### 2.3 Randomization and blinding

After the signing of the informed consent, each participant will then be randomly assigned to IA and SA groups according to a randomization list generated by computerized random-number generator. The injection allocations are printed on cards and inserted into sealed, opaque envelopes. An independent member of the study group, who do not know treatment details, will conduct the allocations.

### 2.4 Participant selection and eligibility criteria

The inclusion criteria require the following:

(1) Adults between the ages of 20 and 70 years;
(2) Patients were diagnosed with primary FS with a normal finding on radiography of the affected shoulder;
(3) passive motion restriction of glenohumeral joint greater than 30 degrees in at least 2 planes of movement;
(4) stage 2 of FS defined by Hannafin and Chiaia with a pain duration of less than 9 months and VAS score ≥3 for shoulder pain.^[6]^

The exclusion criteria require the following:

(1) FS secondary to rheumatic diseases, infectious arthritis, and tumor; infections; rotator cuff disease; osteoarthritis of the glenohumeral joint; fibromyalgia; shoulder fractures; bilateral FS;
(2) patients who received corticosteroids injection on the affected side in the previous 3 months;
(3) inability to understand and cooperate with the investigators or to give valid consent.

### 2.5 Intervention

With the patients sit in an upright position, all injections will be performed by a senior physician with many years of experience in ultrasound-guided injections. The physician is informed about the purpose of study, but patients do not know which injection they will receive. Before injection, adequate iodophor sterilization is carried out around the injection site. 4 mL 2% lidocaine is injected into specified site to anesthetize the skin, followed by 40 mg/mL triamcinolone under ultrasonographic guidance. The IA injection is from the posterior portal and guide into the joint by ultrasonography, while the SA injection is from the lateral portal and guide into the SA space. During the injection, a 25-gauge, 3 cm-long needle is aspirated properly to ensure that the needle is not placed in a blood vessel. Ultrasound imaging confirm the location of the needle. All participants have to stay in the outpatient operating room of hospital for at least 20 minutes to detect and record any acute adverse reactions after the injection including dizziness, skin flushing, and local bleeding. Late adverse events including menstrual disorder, infections, character change, and skin pigmentation will be also checked at follow-up. After the injection, a handbook for rehabilitation programs will be handed out to instruct the patients strengthen functional exercise of shoulder joint. In addition, any additional medication or physical therapies will be also prohibited.

### 2.6 Clinical evaluation

The outcome evaluations will be conducted at 4 time points (baseline, 4, 8, and 12 weeks after the injection) by an independent physical therapist who is blinded to the injection treatments performed. Data will be collected to allow a determination of VAS score for shoulder pain, Constant score, and shoulder passive ROM. The primary outcome is 10-cm VAS score with 0 cm as “no pain” and 10 cm as “the worst imaginable pain”. With patients in the seated position, the passive shoulder ROM, including abduction, forward elevation, external rotation at the side, and internal rotation at the side are measured using a standard goniometer with a scale marked in 1° increments. The examiner moves shoulders slowly and measure 3 times for each shoulder, then the mean angle is used for statistical analysis.

### 2.7 Sample Size

The required sample size in each group are confirmed beforehand by power and sample size calculation. In a previous study conducted by Sun et al,^[12]^ the response within each subject group was normally distributed with standard deviation 3.0 and a significant difference in VAS score at 12 weeks was 2.4. The analysis reveals that a total number of 48 patients (24 patients in each group) are required to provide a power of 80%. The Type I error probability associated with this test of this null hypothesis is 0.05. Accounting for potential loss to follow-up of 20%, we aim to enroll 30 participants per group.

## 3 Discussion

FS is a common shoulder disorder characterized by gradually increasing pain of spontaneous onset and limitations on the range of glenohumeral motions.^[1]^ Although extensive clinical and laboratory studies have been performed, the exact etiology, natural course, and pathophysiology of FS are not yet well understood. FS has been considered as a sequential pathologic process from synovial inflammation to capsular fibrosis.^[16–18]^ An inflammation cascade precipitated by abnormal expression of inflammatory cytokines is thought to be followed by abnormal tissue remodeling and pathologic fibrosis in FS.^[19]^ These heterogeneous stages and the absence of widely-accepted diagnostic criteria have led to controversial results.

Additionally, the nature of the pathologic lesion remains debatable. Typical FS magnetic resonance imaging findings are enhancement and increased thickness of the joint capsule in the axillary recess and rotator interval, including the coracohumeral and superior glenohumeral ligaments.^[20]^ A positron emission tomography/computed tomography study in FS also demonstrated hypermetabolic lesions in the rotator interval, anterior joint capsule, and axillary recess.^[21]^ Although it is clear that the capsule of the glenohumeral joint is involved in the pathogenesis of FS, the results from recent studies revealed that the SA bursa may be a pathologic lesion or a therapeutic target. Lho et al reported that inflammatory cytokines were elevated in the SA bursa of FS, suggesting that the SA bursa may be associated with the pathogenesis of FS.^[22]^ Fluid collection, increased vascularity, or enhancement of the SA bursa on magnetic resonance imaging or ultrasound may be visible in idiopathic FS.^[1]^ In a clinical setting, inflammation of the SA bursa, as well as the joint capsule, can be detected during arthroscopic surgery in patients with refractory FS. Some clinical trials have reported that corticosteroid injection into the SA bursa had an effect similar to that of IA injection in the treatment of idiopathic FS.^[13–15]^ These results suggested that the SA bursa may be a potential pathologic lesion in idiopathic FS. However, there is no clear explanation as to whether the SA bursa is a major or minor clinical lesion. The aim of current study is to compare the short-term effects of corticosteroid injections into different sites, including the IA and SA.

This trial has some limitations. First, the subjects may be exclusively Chinese. Therefore, the data from this clinical trial cannot be applied to other ethnic groups. Second, owing to the small sample size, the results of this study cannot be generalized. Despite these limitations, this trial is expected to provide evidence of proper site of corticosteroid injection for the treatment of FS.

## Author contributions

Yanbiao Wang planned the study design and wrote the study protocol. Yanbiao Wang and Jing Gong reviewed the study protocol. Yanbiao Wang and Jing Gong recruit participants and collect data. Yanbiao Wan wrote the manuscript. All of the authors have read, commented on, and contributed to the submitted manuscript.
